# Learning Experience of Baccalaureate Nursing Students with Challenge-Based Learning in Hong Kong: A Descriptive Qualitative Study

**DOI:** 10.3390/ijerph18126293

**Published:** 2021-06-10

**Authors:** Anson Chui Yan Tang, Meyrick Chum Ming Chow

**Affiliations:** School of Nursing, Tung Wah College, 31 Wylie Road, Homantin, Kowloon, Hong Kong, China; ansontang@twc.edu.hk

**Keywords:** nursing education, challenged-based learning, baccalaureate programme, learning experience

## Abstract

Nursing education has recently adopted challenge-based learning (CBL) to enhance nursing students’ competency in the 21st-century healthcare environment. Previous studies have not yet fully explored nursing students’ perceptions of CBL. This descriptive qualitative study aimed to investigate the learning experience of first-year baccalaureate nursing students engaging with CBL. Videotaped focus group interviews were conducted in a tertiary education institution in Hong Kong, China. The participants recruited for the study included ten Year 1 nursing students enrolled in a public health course delivered using CBL over the study period. The participants included four female and six male students. Five themes were identified: facilitation by teachers, familiarity with CBL, team communication, facilitating metacognitive development, and the application of theories into actual practices. The findings shed light on the potential application of CBL in nursing training as it can foster students’ metacognition, an important attribute for the modern-day workforce. Facilitating theoretical application into practice implies that CBL helps to fill the theory–practice gap that has long been a persistent issue in nursing training. Nonetheless, students encountered frustrating obstacles throughout the learning process, including ineffective group communication, unfamiliarity with the CBL process, and insufficient facilitation by teachers. Better preparation on the part of both teachers and students is critical to ensure that nursing students are gaining optimal benefits from the CBL process.

## 1. Introduction

An increase in connections among communities, countries, and nations is leading to increased complexity in health issues. The current approach to managing health issues has reached a bottleneck; consequently, stakeholders are urging institutions to develop health interventions which can appropriately and efficiently address the complex nature of health issues in the 21st century going forward [1]. National health and nursing agencies have proposed that the future nursing workforce will be highly skilled not only in their own profession but also—equally important—in other generic soft skills, especially multidisciplinary collaboration, innovation, and information literacy, to meet the future needs of the healthcare environment [1,2,3]. Challenge-based learning (CBL), first proposed by Apple, Inc. (2008), is devoted to developing the essential soft skills of the 21st century such as multidisciplinary collaboration, creativity, and technology literacy [4]. Echoing the national recommendations for the expected attributes of future nurses, this study applied CBL to an undergraduate nursing programme in a tertiary education institution in Hong Kong to help nursing students acquire the generic skills essential for the future workplace. This paper reports the learning experience of nursing students who have experienced CBL.

**Figure 1:**
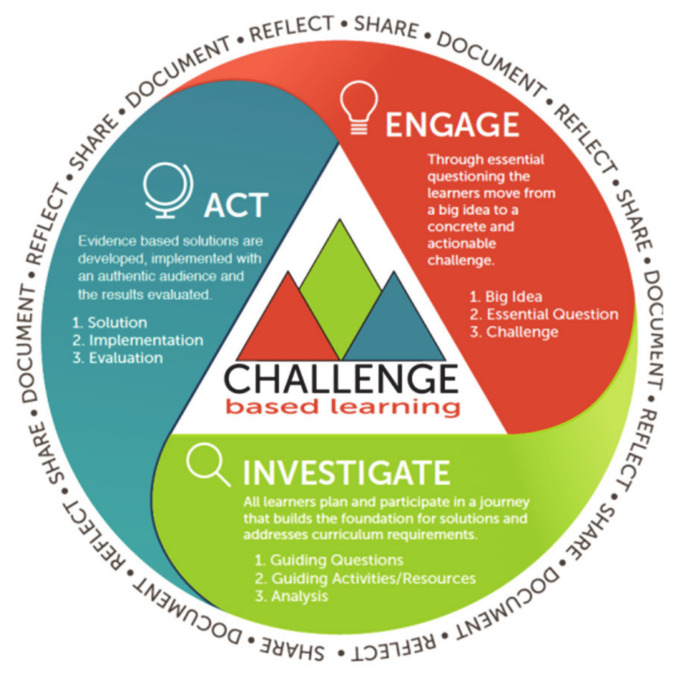
Challenge Based Learning Framework (CBL; Adopted from Nichols et al., 2016, p. 11) [7].

CBL represents a type of experiential learning that involves the processing of knowledge and skills through experience, reflections, experimentation, and application when engaging in learning activities [5]. It creates a learning environment that offers tremendous opportunities for students to explore an issue and create and implement new ideas as deep, wide, and innovative as possible. As Tang and Chow (2020) [6] explained, students must work in a large group which comprises 10 to 13 students, in contrast to the smaller number (i.e., 6–8 students) in a conventional project group, to complete a CBL project according to the three phases of the CBL framework [7]. In Phase 1 (engage), the student group identifies an issue (challenge) relevant to the community in a global health context (big idea). In Phase 2 (investigate), the students go on to analyze the identified issue, brainstorm strategies, and identify appropriate resources to tackle it. During the process, they search for information and consult with relevant experts in the community. These activities allow them to arrive at an eventual solution. In Phase 3 (act), the team implements the solution in the community and then evaluates its outcome (s). Lastly, students disseminate the solution and findings on the Internet such as Facebook, Instagram, and YouTube to share their work with their classmates and the community [7]. Figure 1 outlines the process of CBL.

Existing experiential learning approaches, such as simulation or practicum, largely frame students’ learning experience by providing scenarios or tasks to work on. In contrast, CBL enables students to learn with the greatest degree of autonomy in determining their learning experience. Under this method, students can freely choose a global issue and a corresponding topic without the constraint of pre-existing rules and conditions that teachers have defined. In addition, the composition of the project group involves not merely classmates, as in conventional projects, but also considers relevant expertise outside the classroom. All these key features of CBL, which are not found in other learning approaches adopted in nursing education, are believed to be conducive to help students acquire the desirable soft skills of the 21st century [4]. Some quantitative studies have suggested that CBL can foster students’ soft skills, such as problem-solving skills and creativity [8,9]. In the field of nursing in particular, Tang and Chow (2020) conducted a quasi-experimental study to investigate the effect of CBL on nursing students’ approaches to learning. Their findings support the notion that CBL could facilitate deep learning in nursing students [6].

Tertiary students nowadays are heavily influenced by technology. They tend to be tech-savvy, enjoy diversity in assignments, expect a flexible work environment, and are accustomed to working with people, whether in person or online, instead of working individually [10,11,12]. Accordingly, CBL seems to be an appropriate learning approach to conform to today’s tertiary students’ learning habits and leverage their strengths to maximize their learning potential [13]. A handful of surveys have reported that both teachers and students have positive attitudes towards CBL and perceive CBL as an educational approach beneficial to their learning [8,14,15]. Nonetheless, implementing CBL can be problematic for students who are used to tightly structured lives since birth, especially in Asian cities such as Hong Kong. In this culture, parents organize and vigilantly supervise their children’s daily lives by arranging all sorts of activities for them [16]. They may become comfortable living under established rules and lack the capability of mastering and managing uncertainties in their life without a lot of guidance and supervision [17].

As the current literature on the learning experience of nursing students in a CBL framework is scarce especially in Asian context, the authors conducted semi-structured focus group interviews to vividly describe the lived experience of Hong Kong nursing students using CBL in this study. The findings can provide insightful information to nurse educators about CBL from the students’ perspective. Nurse educators may make use of the reported findings to modify CBL integration into their curricula to address the learning needs of their students regarding the application of CBL in nursing education.

## 2. Materials and Methods

### 2.1. Design and Setting

This was a descriptive qualitative study using semi-structured focus groups to explore the learning experience of Hong Kong nursing students using CBL. It was part of a larger research effort examining the effectiveness of CBL for baccalaureate nursing students. This study was conducted at a tertiary education institution in Hong Kong. The focus group interviews were conducted in a classroom of the institution.

### 2.2. Participants

Nursing students studying in a baccalaureate nursing programme were the target population. The accessible group comprised 199 Year 1 nursing students who had enrolled in a public health course, which had adopted CBL as a major learning approach, over the study period. A mass email was sent to the Year 1 students to invite them to participate in a group interview by the end of the course. A convenience sample of ten participants was recruited and they all agreed to be interviewed. The research team stopped recruiting further due to data saturation.

### 2.3. Intervention

CBL was incorporated into the public health course offered in Semester 2 of the first year of the programme. Over 14 weeks, the students received one 2-h didactic lecture, which discussed relevant theories and information related to public health, and one tutorial designed with CBL for group projects every week. The tutorials were run according to the CBL framework as well as the classroom guide published by Apple, Inc. (2011) [18]. The project guideline was explained and distributed to the students in the first tutorial to familiarize them with the CBL process and project requirements. No guiding questions were given to students except the description of the CBL framework [7] and some examples of global issues (e.g., health inequalities, ageing, mental health). Table 1 illustrates a typical learning plan for the tutorials designed with CBL in the public health course over the 14 weeks.

The students organized themselves into groups of 12–13 to complete the project. The students also identified and invited relevant people in the community to provide input to design, implement, and evaluate the solution. The planned solution was applied to the target people in the community in Weeks 9–12. Group presentations to the whole class were conducted during Week 14.

During tutorial, each group shared their progress according to the pre-specified timeline (Table 1) with the respective tutors. The major roles of the tutors in the tutorials were: (1) to monitor project progress by means of a weekly/biweekly written progress report and (2) to ensure each member in the project group had sufficient participation in the project to avoid freeloaders. In addition, if at any time a group had unresolved conflicts, the tutor would step in to facilitate resolution. Otherwise, all ideas about the project and division of work were solely determined by the student groups themselves.

Prior the commencement of the course, teachers in the teaching team of the public health course attended an in-house training workshop, which explained the principles and process of CBL, and the techniques of supervising projects designed with CBL. The workshop was conducted by the course coordinator, who had received training in CBL and got tremendous experience of designing courses with CBL. Demonstration and return demonstration were provided to ensure the teachers supervising the projects could managed group conflicts, if they arose, in an appropriate manner.

### 2.4. Data Collection

The principal investigator conducted three group interviews, each involving three to four participants. Written consent was gained from the participants prior the start of the interviews. The principal investigator, who was not involved in teaching the public health course, was the interviewer. The interview began with ice-breaker questions, followed by five guiding questions focusing on the research objective:

1. Was the level of support adequate during the learning process?
2. What did you like the most about this learning approach?
3. What did you like the least about this learning approach?
4. How did this learning approach affect your learning?
5. Is there anything about this learning approach you want to modify to facilitate your learning?

The interviewer asked probing questions to encourage interviewees to elaborate on their answers, their experiences and their feelings towards CBL. All interviews were videotaped, and each interview took about one hour.

### 2.5. Data Analysis

Thematic analysis was used to code, categorize, and analyze the data in accordance with Braun and Clarke’s (2006) six phases [19]. The research assistant first transcribed the videotapes. Next, the principal investigator counterchecked the transcriptions against the videos to ensure that the transcriptions were consistent with participants’ non-verbal behaviour during interviews. The transcribed verbatim text was then returned to each participant for confirmation of the accuracy of the transcription.

The principal investigator first read the transcripts several times to get familiar with the content. Relevant meaningful sentences corresponding to the interview questions were extracted and coded in a systematic manner. The codes were compared to identify similarities and differences. An initial thematic map was formulated by subsuming initial codes under potential themes and sub-themes. The initial thematic map was then compared with the coded data again to look for discrepant interpretations. To validate the analysis and ensure accuracy, the co-investigator reviewed the analysis.

### 2.6. Ethical Considerations

The participants were informed that their conversations in the interviews were blinded to the course teachers and not linked to their course performance. Participation was voluntary, and those who declined to join the focus group interview or discontinued participation during the study did not receive penalties of any kind. Ethics approval was obtained from the self-financing institution before data collection started.

## 3. Results

Ten nursing students (four male, six female) participated in the interviews. All were Year 1 students in the programme and had no previous experience with CBL. From the interviews, five themes emerged regarding the essential elements of learning using CBL as well as how CBL can facilitate learning: facilitation by teachers, familiarity with CBL, team communication, facilitating metacognitive development, and the application of theory in practice.

### 3.1. Facilitation by Teachers

The idea of ‘facilitation by teachers’ refers to guidance from tutors, namely the teaching staff who led the CBL tutorials. This includes a sufficiency of quality guidance and teachers’ understanding of CBL. All the interviewees agreed that tutors played a significant facilitative role in the smooth completion of their projects. One of the participants stated:

“*The tutor should give us a lot of guidance. We need to identify a challenge and the corresponding essential questions, then look for appropriate measures to solve the problem. We may come up with several ideas; we need the tutor’s advice to identify the most appropriate approach/idea to solve the problem*.”

The participants perceived quality of guidance as the most essential component of the facilitation. In the opinion of another participant:

“*[Success] really depends on the tutor’s support and guidance it heavily depends on the quality of his/her guidance on the direction of the project we won’t be off track if he/she guides us to a right direction to understand what students don’t understand and what problems we face.*”

However, the participants felt that some tutors did not seem to understand the CBL process well and gave limited or inconsistent guidance. One participant described this shortcoming as follows:

“*We didn’t understand this process. There was a guideline showing the CBL process and requirement, the tutor just asked us to follow that [CBL] framework to complete the project, but they didn’t clearly explain to us what was expected in each stage in detail...the tutors seemed having different understandings about the requirements.*”

### 3.2. Familiarity with CBL

‘Familiarity with CBL’ reflects the students’ understanding of the CBL process and its intended influence on their learning in a course. The participants reported that CBL was a new concept to them, and the project requirements were far more demanding than those for the projects they were used to working on. They found the process difficult and frustrating. One participant summarized the whole group experience in the following perception:

“*I got lost...CBL is new to me, I didn’t know why we needed this approach...It is a rather complicated process, we needed to spend far more time to search information [as compared with conventional group projects].*”

Another participant added,

“*CBL requires us to develop a topic by ourselves, we need to do all the things by ourselves [without intensive guidance from tutors] ...it is very difficult and frustrating.*”

### 3.3. Team Communication

‘Team communication’ refers to how students adjusted their communication strategies in response to CBL. In this study, the participants realized the need to modify their communication and division of work according to the different natures and requirements of CBL as compared to a conventional project approach. One participant felt that the large group size created logistical and communication issues, saying,

“*More than ten students in a group...[I] don’t like so many students in a group...In term of communication, our group is too large, [we] are often unable to meet all at a time because of different class schedules...it is difficult to communicate the work done by individual member efficiently and effectively. We need to repeat the same message many times...We need to readjust our communication strategies in such a large group...effective communication in a large group itself is a challenge to us.*”

The students realized that they needed to adjust their communication strategies for the CBL group project. They could not apply the same strategies used in a conventional group project on a CBL-based project. One participant explained,

“*Our group worked together without dividing work. We met in a computer room and did the project together...it is difficult to divide work among members like what we were used to do in a conventional group project...We usually met and worked on it together...discussed what we needed and filtered out those not appropriate because once the first step gets wrong, the rest followed will go wrong.*”

### 3.4. Facilitating Metacognitive Development

This theme describes how students acquired a comprehensive deep-thinking approach to manage an issue. Metacognition refers to cultivation of a habit to observe one’s thinking process, self-monitor one’s efficiency and effectiveness of learning, and make modifications accordingly [20,21]. Students with strong metacognition can critically analyze an issue and information from different perspectives. Most participants agreed that CBL provided a frame for them to resolve an issue logically and holistically. A participant described his experience as follows:

“*[CBL] is comprehensive...we can complete a project comprehensively...we don’t have such framework in previous group projects which were in fact very loose. Now we know well...starting to look for a problem in a community, identifying appropriate expertise to support the problems and solution...then implementation, we think of some solutions to resolve the issue, to tell the public that the issues could be solved in such way...very holistic.*”

In addition, the students maintained that the CBL framework modified their thinking approach; they believed they would apply the same thinking approach to other assignments. As a result of experiencing CBL, students came to understand ‘know-how’ when facing a problem. They are now able to transfer the same thinking techniques into other situations. A participant expressed affirmatively that:

“*The largest gain after going through this process is we know how to pick out core issues from a health problem. When I did the project in the pediatric course...I worked on short-sightedness...I applied the same thinking process as in the stage ‘Engage’ of the CBL framework to identify the high-risk group by asking essential questions and looking for relevant evidence to support it.*”

### 3.5. Facilitating Application of Theory in Practice

This theme describes how the solid experiences gained in the CBL project enabled students to understand the necessity of tailor-making interventions for the target population based on the latter’s uniqueness while increase the student’s awareness of the actual needs of the community. Such an understanding helps students to apply theoretical concepts in real practice. A participant reflected,

“*It helped me think from different perspectives. In the past, I just learned a disease from [books] and didn’t consciously analyze the information to justify the need of the assigned topic to the community/target people. Now, the identified topic is an actual need of the community.... CBL allows me to understand and analyze the actual needs of our society that I learned in lectures. I pay more attention to the need of the society now. I won’t just focus on fulfilling the assignment...I now know how to identify the actual health needs of our society.*”

Another participant responded:

“*Knowing how to analyze the information...and how to identify the target group for a health issue...we need to tailor-make activities corresponding to the characteristics of different target groups...for example, if it is adolescent, we will use interactive approach to attract them to join our activities...if we work on a topic related to middle-aged people such as cardiovascular disease, I will use another approach to arouse their interest towards our activities.*”

## 4. Discussion

The findings describe the perceptions of nursing students who encountered CBL in an Asian context. The particular theme presented in this paper relating to the facilitation of metacognitive development echoed the results of Tang and Chow (2020) [6], who reported that CBL can facilitate deep learning in nursing students. Metacognitive ability is not a new concept in nursing. Nurse educators have been seeking potential pedagogies such as problem-based learning (PBL) and simulation to promote nursing students’ metacognitive abilities [22]. Similar to other experiential learning approaches, CBL could bridge the theory–practice gap that remains a continuing problem for nursing students and newly qualified nurses [23,24]. The participants agreed that tackling an authentic health issue enabled them to familiarize themselves with the characteristics and understand the actual needs of different people in a society. CBL may increase their social awareness and realization of the roles of nurses in different settings. These experiences may also allow them to see the direct relevance of their learning to their future career, which may further enhance their motivation to learn [25,26].

Three out of five themes describe the difficulties the students encountered: team communication, familiarity with CBL, and teachers’ facilitation. Previous studies exploring students’ learning experience with new learning approaches have reported similar findings [27,28]. Harris and Kloubec (2014) [27] found that students felt frustrated by a problem-based learning (PBL) project because they did not always grasp the project requirement. In addition, the target participants in this study were Asian students who had seldom been exposed to such highly flexible conditions in previous learning situations or in life [16,17], possibly adding extra pressure on them as it was the first time they were tasked with managing an issue fully independently.

Effective communication within a project team was a major concern that the students raised regarding working in a large group. Previous studies, although involving smaller-sized groups, have reported the same problem [27]. The quality of group interaction was found to play a pivotal role to the learning outcomes because both high and low achievers were able to benefit from a high-quality group process [29]. It seems that the participants in this study did not work well because they lacked the essential elements of high-quality group interaction such as good division of work and effective communication skills [30,31]. A CBL project provides an opportunity for students to try out communication strategies required for effective group communication as they will face the same conditions when they work as professional nurses in a diverse healthcare environment in the future. Early exposure to situations that can polish communication and collaboration can help students adapt to their future workplaces more quickly and smoothly [32].

The participants noted that the CBL project would have been more enjoyable and would have reduced their frustration if they had known more about the CBL process from the beginning. They also felt a need for the tutor to have provided more explicit guidance at each phase. Evidence shows that a tutor’s attitude and understanding of an educational approach are contributory factors to learners’ satisfaction and performance [33,34]. In this study, while the course coordinator was experienced, the teachers were facilitating CBL project groups for the first time. Although they had received training before the study, they still had no actual experience in teaching using CBL. Their perceptions of their own inexperience or less than full commitment to CBL might have undermined their ability to coach groups effectively. It is possible that the received training might not have been sufficient to address their educational needs and so might have impeded them in performing competent facilitative roles in CBL tutorials.

This study presents several limitations that may affect trustworthiness. First, the study was conducted in Hong Kong, which may have a different educational culture compared to other Asian countries. The group composition was relatively homogenous, comprising only students in the same class and a few relevant people from the community. Some groups included one to two students from higher years who had previously dropped the course. The homogeneous composition of the project groups might have limited the participating students’ experience in a multidisciplinary group. Thus, a more heterogeneous project group may fully reflect the full spectrum of a team interaction experience. As mentioned, the tutors were coaching CBL project groups for the first time. Their inexperience in coaching CBL group work might have affected students’ perceptions of this learning pedagogy.

Better preparation for both students and tutors is paramount to students’ positive learning experience. It is recommended that the course coordinator should thoroughly orient the students at the start of the course, including a clearly defined purpose and expectations at each phase of the CBL process to help alleviate student frustration towards this learning approach. Also, before the initial CBL tutorial, the tutors could spend time training students to work effectively in groups, including role assignment, communication, and conflict management [27]. From the teachers’ side, more comprehensive training for teachers would be helpful to ensure that they fully understand the processes of CBL, especially if they are teaching CBL for the first time; they are committed to it; and they know how to coach groups effectively. In addition to providing training and debriefing sessions to nurse educators, exploring nurse educators’ perception (s) or attitudes involving a new approach in training nursing students appears to be valuable.

## 5. Conclusions

This is the first study exploring nursing students’ perceptions of CBL in an Asian context. The participants reported that CBL facilitated their metacognitive development and application of theoretical knowledge in practice. However, three out of the five themes described the difficulties the students encountered while conducting group projects following the CBL approach. Team communication, familiarity with the CBL process, and the tutors’ facilitation are the key areas where students expressed frustration. Given the value of CBL in promoting students’ metacognitive thinking and resolving the continuing theory–practice gap in nursing education, CBL should be promoted in nursing education. However, nurse educators should pay special attention to both students’ and tutors’ concerns regarding their understanding of the purpose, requirement, process of CBL and related topics in preparation for a successful CBL process. Furthermore, tutors’ teaching experience with CBL is an area worth exploring as the results can provide insight for stakeholders in developing fit-for-purpose training for tutors.

## Figures and Tables

**Table 1:** Learning plan for group project in the public health course over the 14 weeks.

**Learning Task(s)**	Project design & planning Project implementation & evaluation
**Assessment** **Component(s)**	Project presentation Individual reflective paper
**Group size**	12–13 students
**Project schedule**	
**Semester week**	
**Week 1–4**	Stage 1—Engage
	Select Big Idea such as mental health, infection control, obesity Identify the essential question and the challenge Conduct needs assessment
**Week 5–7**	Stage 2—Investigate
	Devise solution with appropriate strategies based on site assessment/interview(s) with relevant parties and experts/information in the literature
**Week 8–14**	Stage 3—Act
	Formulate implementation and evaluation plan Deliver the solution & evaluate the solution outcome(s) Disseminating the project via social media platforms such as You-tube, Facebook Reflection

## Data Availability

Data available on request.
